# Elution with 1,2-Hexanediol Enables Coupling of ICPMS with Reversed-Pase Liquid Chromatography under Standard Conditions

**DOI:** 10.1021/acs.analchem.2c01769

**Published:** 2022-06-06

**Authors:** Bassam Lajin, Joerg Feldmann, Walter Goessler

**Affiliations:** † Institute of Chemistry, Analytical Chemistry for the Health and Environment, University of Graz, Universitaetsplatz 1, 8010 Graz, Austria; ‡ Institute of Chemistry, TESLA (Trace Element Speciation Laboratory), University of Graz, Universitaetsplatz 1, 8010 Graz, Austria

## Abstract

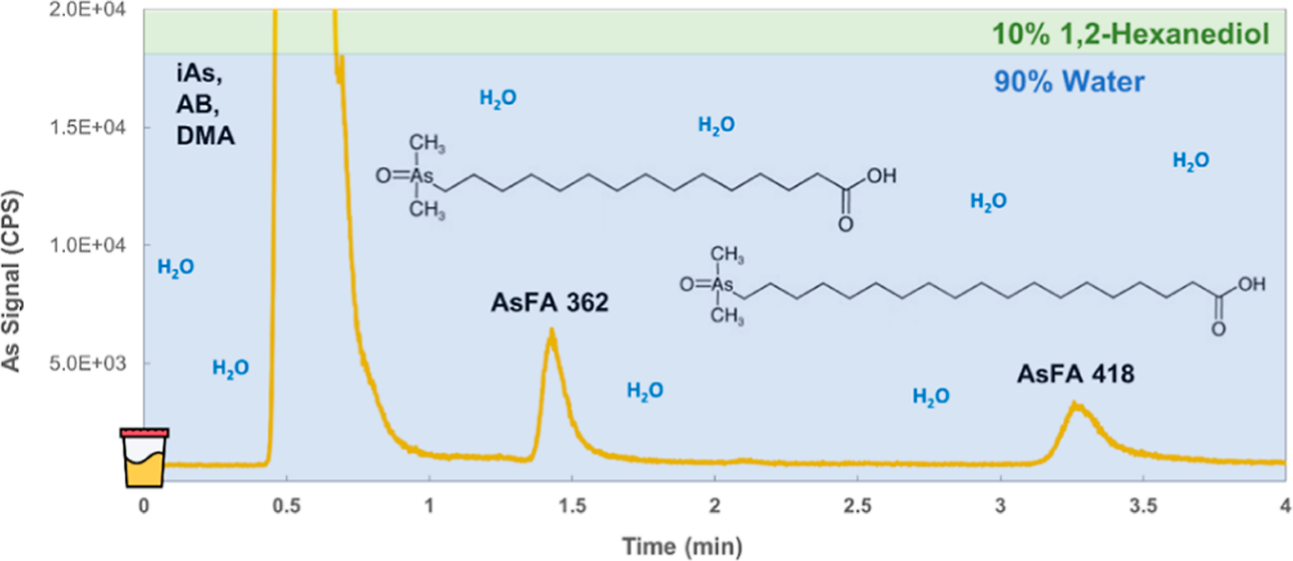

The inductively coupled plasma mass spectrometry (ICPMS) has been attracting increasing attention for many applications as an element-selective chromatographic detector. A major and fundamental limitation in coupling ICPMS with liquid chromatography is the limited compatibility with organic solvents, which has so far been addressed via a tedious approach, collectively referred to as the “organic ICPMS mode”, that can decrease detection sensitivity by up to 100-fold. Herein, we report 1,2-hexanediol as a new eluent in high-performance liquid chromatography–ICPMS which enables avoiding the current limitations. Unlike commonly used eluents, 1,2-hexanediol was remarkably compatible with ICPMS detection at high flow rates of 1.5 mL min^–1^ and concentrations of at least 30% v/v, respectively, under the standard conditions and instrumental setup normally used with 100% aqueous media. Sensitivity for all tested elements (P, S, Cl, Br, Se, and As) was enhanced with 10% v/v 1,2-hexanediol relative to that of 100% aqueous media by 1.5–7-fold depending on the element. Concentrations of 1,2-hexanediol at ≤30% v/v were superior in elution strength to concentrations at >90% v/v of the common organic phases, which greatly decreases the amount of carbon required to elute highly hydrophobic compounds such as lipids and steroids, enabling detection at ultra-trace levels. The proposed approach was applied to detect arsenic-containing fatty acids in spiked human urine, and detection limits of <0.01 μg As L^–1^ were achieved, which is >100-fold lower than those previously reported using the organic ICPMS mode. Nontargeted speciation analysis in *Allium sativum* revealed the presence of a large number of hydrophobic sulfur-containing metabolomic features at trace levels.

## 1 Introduction

The employment of the inductively coupled plasma mass spectrometry (ICPMS) as an element-selective detector for liquid chromatography has gained increasing popularity in previous years,^1,2^ particularly since the introduction of the tandem mass spectrometry (MS/MS) technology to the technique,^3^ which effectively resolved polyatomic interferences.^4,5^ Coupling chromatography with an element-selective detector enables a unique approach to chemical speciation analysis, involving comparative and simultaneous detection and quantification of chemical forms of multiple elements^6^ as well as serving as a tool for the discovery of novel compounds of environmental, biological, or industrial origin,^7−10^ particularly through simplifying molecular metabolomic data in nontargeted analysis.^11,12^

Reversed-phase liquid chromatography is by far the most common separation mode but involves the employment of mobile phases containing high percentages of an organic solvent. Apart from the general environmental aspects, the use of high quantities of organic solvents presents a specific challenge for chromatographic detection with ICPMS due to the known low tolerability of carbon by the inductively coupled plasma, resulting in plasma shutdown at high carbon load. The general intolerability of carbon by the inductively coupled plasma has been a well-recognized and thoroughly investigated subject.^13,14^ For chromatographic speciation analysis using an ICPMS detector, this obstacle has so far been partly overcome by applying modifications in terms of instrumental setup and experimental conditions,^15−17^ which can be collectively referred to as the “organic ICPMS mode”.

In current practice, the organic ICPMS mode involves combinations of the following: (1) using oxygen as an optional gas to help volatilize carbon and prevent its buildup on the cones, which is associated with decreased interface and analyzer pressure, signal drift, and clogging of the sampler and skimmer cones; (2) replacing the Cu/Ni cones with the more expensive Pt cones, which is recommended under continuous operation with oxygen as an optional gas to prevent corrosion; (3) mobile phase flow rate splitting (usually 1:5–1:10) and/or postcolumn dilution; (4) employing sub-zero temperature for the spray chamber; and (5) using a plasma torch with a 1.5 mm injector rather than the standard 2.5 mm injector.

Many of the above components of the organic ICPMS mode can negatively impact sensitivity, not only through flow splitting/postcolumn dilution but also through the addition of oxygen to the plasma which may decrease sensitivity, for example, by increasing the formation of competing oxides and polyatomic species. Using a narrow injector plasma torch increases carrier gas velocity and therefore shortens sample residence time in the plasma, which can negatively impact drying, decomposition, and ionization efficiency.

One case exemplifying the consequences of the organic ICPMS mode is the speciation analysis of arsenolipids, where detection limits of 1.0–10 μg As L^–1^ are typically reported,^18−20^ which is >100-fold higher than typical detection limits reported for low-molecular-weight hydrophilic arsenic species not requiring the ICPMS organic mode (0.005–0.03 μg As L^–1^).^21−23^ The latter low detection limits are otherwise achievable for the arsenolipids with molecular mass spectrometric detection,^24^ which is often used in combination with ICPMS detection for identifying novel species in this class of actively explored arsenic compounds.^17,25^ However, the current gap in detectability between the two techniques renders the powerful combination of elemental and molecular MS incapable of identifying new species in this class of compounds at such low concentration levels, where ICPMS is the limiting component.

Furthermore, the increased complexity and reduced convenience associated with the organic ICPMS mode has apparently been a deterring factor in many nontargeted analysis studies where low organic content mobile phases were employed,^26,27^ and for some elements, this has likely lead to a gap (i.e., low column recovery) between the total elemental content and the sum of the individual species detected.^28−30^ It is plausible that the inclusion of mobile phases with higher elution strength in nontargeted screening speciation analyses using high-performance liquid chromatography (HPLC)-ICPMS on a routine basis would enable the identification of more novel compounds.

Organic solvents are known to show a wide variation in their tolerability by the inductively coupled plasma depending on a set of key physicochemical properties including boiling point, vapor pressure, viscosity, surface tension, and density.^13^ As a proof of concept, we previously introduced dimethylcarbonate as a new solvent in HPLC-ICPMS,^31^ which was found to offer superior elution strength to commonly used organic solvents such as acetonitrile and methanol, but the utility of this solvent was limited to concentrations ≤10% v/v, and these concentrations were insufficient to elute highly hydrophobic compounds (e.g., lipids).^31^ The aim of the present work was to find alternative organic solvents that can show high compatibility with ICPMS detection and provide exceptionally strong chromatographic elution at lower eluent concentrations in order to enable coupling reversed-phase chromatography with ICPMS detection under default conditions and standard experimental setup without the need to employ any of the components of the organic ICPMS mode, which would have the advantage of increasing the detection capability of the technique while rendering analysis more convenient.

We herein introduce 1,2-hexanediol as a new eluent to speciation analysis via liquid chromatography coupled with ICPMS detection, and we examine its properties and highlight its advantages, remarkable tolerability by the plasma, and potential to eliminate the need for the organic ICPMS mode and its associated disadvantages.

## 2 Experimental Section

### 2.1 Chromatographic Separation

All chromatographic investigations were performed using the reversed-phase column Zorbax Eclipse Plus C18, 50 mm × 2.1 mm i.d., 1.8 μm (Agilent Technologies, Waldbronn, Germany). A short column length was chosen to enable the observation of the chromatographic behavior and the calculation of retention factors up to *k* = 50 within reasonable retention times (<30 min) in all experiments. It is important to keep in mind that chromatographic retention is correctly measured by the retention factor, which is independent of the column length, rather than the retention time. The retention factors (capacity factors) were calculated based on the compound retention time (*t*_R_) and column void time (*t*_0_) using the formula *k* = (*t*_R_ – *t*_0_)/*t*_0_ and used as a measure of retention throughout the study. The column void time (*t*_0_) was estimated to be 0.55 min based on the retention time of the unretained sulfate anion.

The following general chromatographic conditions were employed for all experiments unless otherwise stated: mobile phase flow rate: 0.25 mL min^–1^; column temperature: 50 °C; injection volume: 1.0–3.0 μL; and mobile phase composition: formic acid 0.1% v/v (ACS grade, purity >98%) with variable contents of the different organic solvents (1,2-hexanediol, methanol, acetonitrile, or isopropanol). Elution was performed isocratically, and the mobile phases were prepared via online mixing of a 2.0% v/v solution of formic acid, purified water produced in-house using a Milli-Q water purification system (18.2 MΩ cm, Merck Millipore GmbH, Vienna, Austria), and pure acetonitrile, methanol, or isopropanol. A solution of 1,2-hexanediol was prepared offline at a concentration of 30% v/v in purified water, mixed, and sonicated for 10 min. The resulting aqueous solution of 1,2-hexanediol was then treated similarly to the other pure organic solvents, as described above. Chromatographic reagents and solvents, including 1,2-hexanediol (purity 98%, CAS-Number 6920-22-5), were purchased from Sigma-Aldrich (Steinheim, Germany).

**Figure 1:**
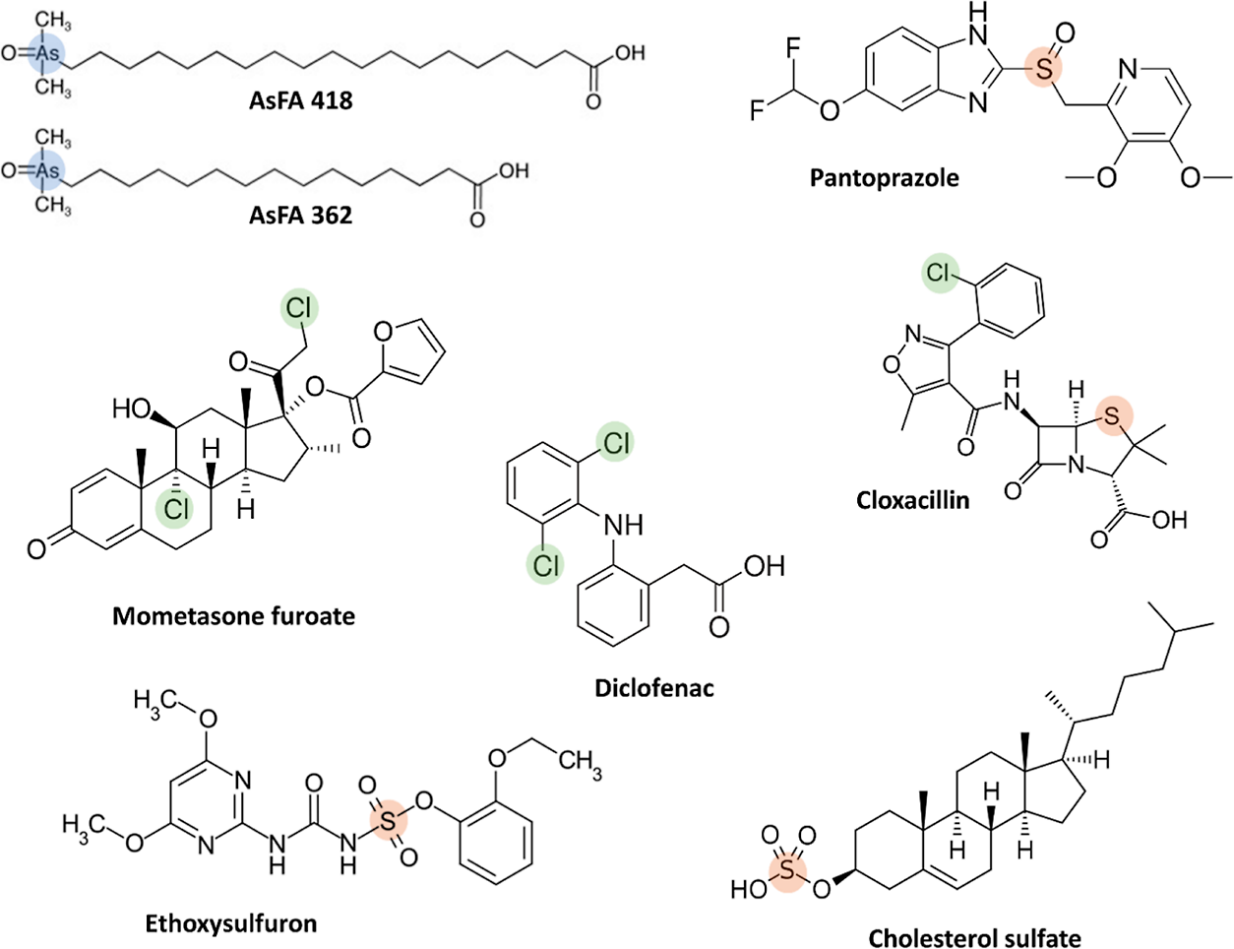
Chemical structures of the hydrophobic model compounds chosen in the current study (Log *P* 2.4–8.2). ICPMS/MS detection was based on the highlighted heteroatoms.

A group of hydrophobic compounds with a LogP within the range of 2.4–8.2 (computed using XLogP3 3.0^32^) were selected as model compounds (Figure 1) in order to investigate the elution properties of 1,2-hexanediol in comparison with those of the commonly used organic solvents methanol, acetonitrile, and isopropanol. The selected compounds are of medicinal/environmental interest and detectable via ICPMS through a heteroatom. Standard solutions were prepared in pure methanol at concentrations of 20–50 mg element L^–1^, unless otherwise stated, and injected onto the column using the conditions described above.

### 2.2 Chromatographic Detection

ICPMS/MS detection was performed using an Agilent 8900 ICPQQQ system coupled with an Agilent 1100 HPLC system. The ICPMS/MS system consisted of an AriMist polyether ether ketone (PEEK) nebulizer (maximum nebulizer gas flow rate: 0.8 L min^–1^), a glass double pass spray chamber (cooled at 2 °C), a nickel/copper sampler and skimmer cones, and a quartz plasma torch with an injector inner diameter of 2.5 mm. The use of oxygen as an optional gas or flow splitting/postcolumn dilution was deliberately avoided in all experiments with 1,2-hexanediol.

The ICPMS/MS detector was operated using the following parameters: RF power: 1550 W; plasma gas: 15.0 L min^–1^; auxiliary gas: 0.9 L min^–1^; RF matching: 1.3–1.8 V [depending on the concentration of 1,2-hexanediol in the mobile phase (0–30% v/v) at a 0.25 mL min^–1^ flow rate]; sampling position (sampling depth): 5.0 mm; nebulizer gas flow rate: 0.65 L min^–1^; makeup gas (argon) flow rate: 0.25–0.45 L min^–1^ (used to yield a total carrier gas flow of 0.9–1.1 L min^–1^ depending on the organic content; lower organic content required a higher total carrier gas flow rate for optimum sensitivity); optional gas: 0.0%; nebulizer pump speed (for drainage): 0.50 rps (ca. 2.0 mL min^–1^); and S/C (spray chamber) temperature: 2 °C. Chlorine was detected in the hydrogen mode (H_2_ flow rate: 3.5 mL min^–1^) by monitoring the transition *m*/*z* 35 → 37 (as ^35^Cl^1^H_2_^+^), and all other elements in the oxygen mode (O_2_ flow rate: 0.3 mL min^–1^) by monitoring the transitions corresponding to *m*/*z* M^+^ → M^+^ + 16.

Chromatographic experiments involving mobile phases containing >10–25% of methanol, acetonitrile, or isopropanol could not be performed with ICPMS detection under the standard conditions described above due to plasma instability. For simplicity, an Agilent 1260 spectrophotometric detector was employed to investigate these solvents, using chromatographic conditions identical to those described above with ICPMS detection. Furthermore, confirmation of the elution patterns displayed by the most hydrophobic compounds included in the study (cholesterol sulfate and arsenic-containing fatty acids) was performed using a molecule-selective detector (Agilent triple quadrupole Ultivo ESIMS/MS system) by monitoring *m*/*z* 363 and 419 for the arsenic-containing fatty acids AsFA 362 and AsFA 418, respectively, in the positive mode, and *m*/*z* 465 for cholesterol sulfate in the negative mode using the following source settings: nebulizer gas temperature and flow rate: 350 °C and 10 L min^–1^, respectively; sheath gas temperature and flow rate: 400 °C and 12 L min^–1^, respectively; nebulizer pressure: 35 psi; and capillary voltage: 3000 V.

## 3 Results and Discussion

### 3.1 Selection of 1,2-Hexanediol

The degree of plasma tolerability for organic matrices depends on various physicochemical properties, most notably, boiling point, vapor pressure, and viscosity,^13^ and the selection of 1,2-hexanediol was based on these properties. In particular, an extremely low vapor pressure of 2.7 Pa at 20 °C (0.02 mmHg) and a high boiling point of 224 °C would be expected to result in low vapor transport and carbon load on the plasma. On the other hand, a high LogP of 0.7^32^ for 1,2-hexanediol would enable higher chromatographic elution strength at lower organic content. The superiority of 1,2-hexanediol becomes clear when comparing the above-mentioned properties with those for the commonly employed solvents methanol, acetonitrile, and isopropanol (see Supporting Information Table S1).

This is not the first report describing the incorporation of 1,2-hexanediol in a mobile phase for reversed-phase liquid chromatography. Li and Fritz reported the addition of 1% v/v 1,2 hexanediol as a chromatographic modifier to improve the separation of hydrophilic organic acids (e.g., formic acid and acetic acid) under spectrophotometric and conductometric detection.^33^ However, the behavior of 1,2-hexanediol as a general chromatographic eluent (i.e., at concentrations >1%) rather than a chromatographic modifier was not previously reported, and its employment with ICPMS detection has not been previously described.

### 3.2 Chromatographic Elution Behavior

A group including highly hydrophobic compounds (up to a LogP of 8.2, computed using XLOGP3 3.0^32^) was selected (Figure 1). These compounds are amenable to detection via ICPMS through the presence of a heteroatom and are relevant to pharmaceutical, biological, and/or environmental applications.

**Figure 2:**
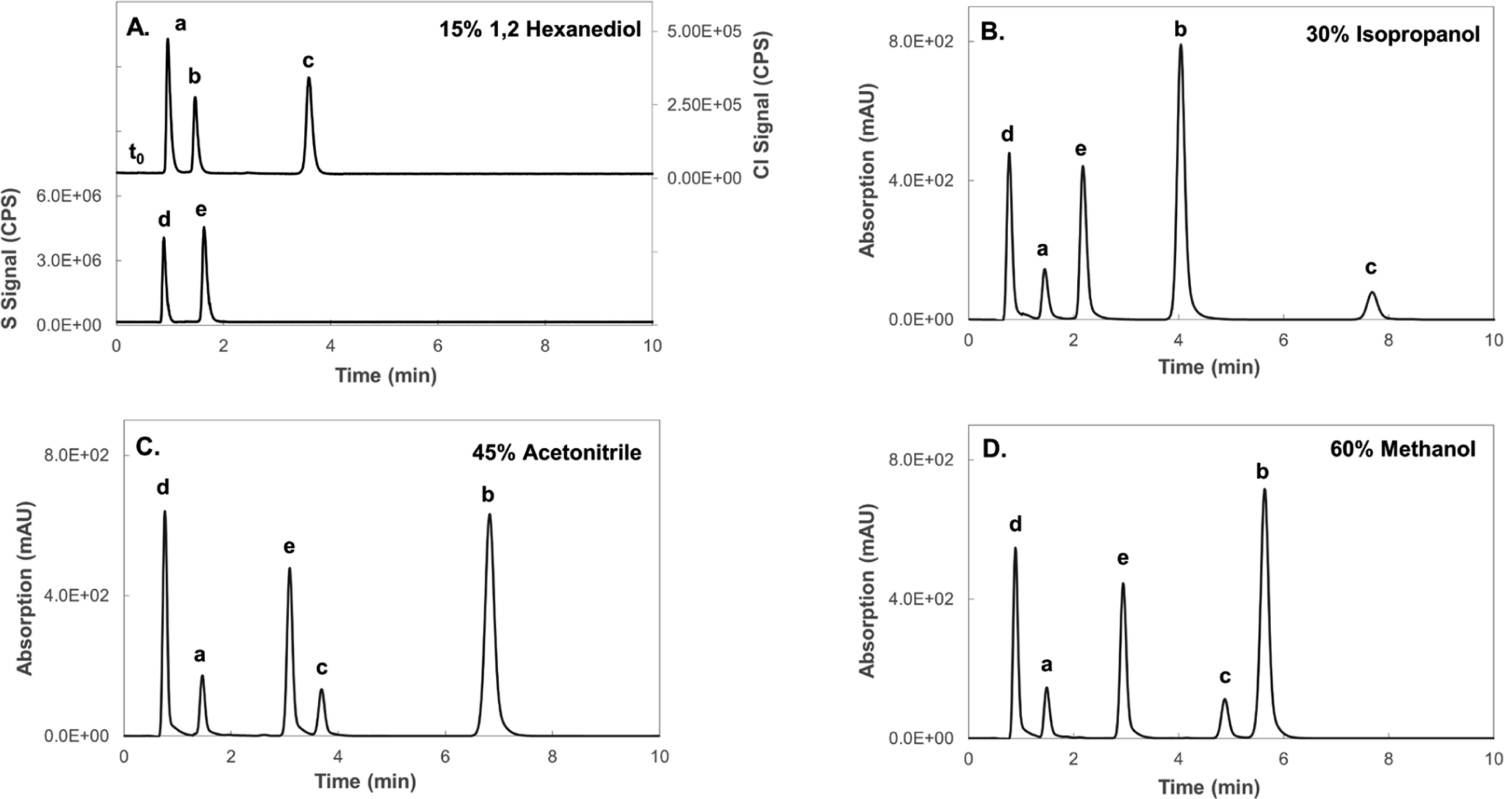
Comparing the elution strength of 1,2-hexanediol (A) with solvents commonly used as eluents in reversed-phase chromatography, namely, isopropanol (B), acetonitrile (C), and methanol (D). Only 1,2-hexanediol was compatible with direct ICPMS/MS detection at the investigated concentration range (for conditions, see the Experimental Section), and therefore, detection with the other eluents was undertaken using a spectrophotometric detector at 254 nm. The column void time is 0.55 min. Note that the selectivity (and peak order) for 1,2-hexanediol is similar to that of isopropanol and differs from that of acetonitrile and methanol. Peak a: cloxacillin; peak b: mometasone furoate; peak c: diclofenac; peak d: pantoprazole; and peak e: ethoxysulfuron.

**Figure 3:**
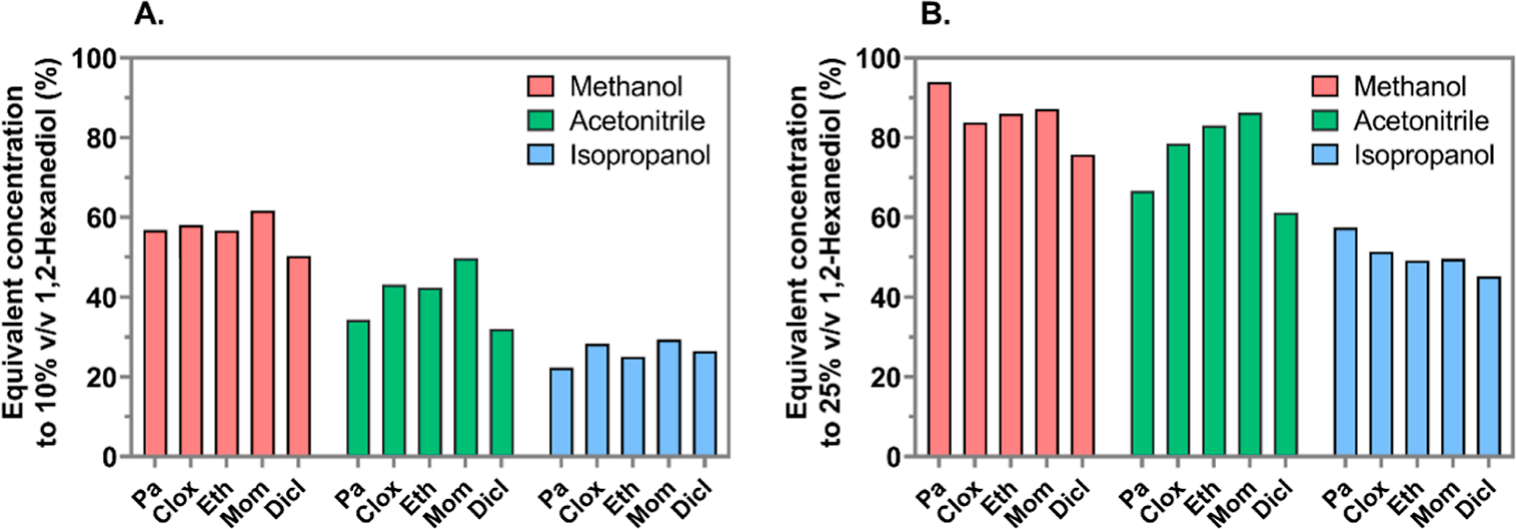
Concentrations of eluents showing comparable elution strength to that of 10% (A) and 25% v/v (B) of 1,2-hexanediol. The values were calculated based on linear regression lines (*r*^2^ = 0.9990–0.9999) constructed by plotting log *k* (retention factor) values against log *C* % (percentage concentration), based on experiments where retention times were recorded under varying organic solvent proportions. Note that for highly hydrophobic compounds (e.g., cholesterol sulfate investigated in the present study), the elution strength of >20% v/v 1,2-hexanediol may not be matched by any concentration of methanol or acetonitrile (see Supporting Information Figure S1). Pa, pantoprazole; Clox, cloxacillin; Eth, ethoxysulfuron; Mom, mometasone furoate; and Dicl, diclofenac.

1,2-Hexanediol showed superior elution strength relative to commonly used solvents even when these were employed at much higher concentrations (Figure 2). Overall, direct experimental data as well as calculations based on the linear regression of the Log *C* (eluent concentration) versus Log *k* (retention factor) relationship revealed that 1,2-hexanediol at concentrations within the range of 1.0–25% v/v can replace methanol, acetonitrile, and isopropanol within the concentration ranges of 20–95, 10–85, and 5.0–60%, respectively (Figure 3). For highly hydrophobic compounds such as cholesterol sulfate, the elution strength of 1,2 hexanediol at >25% v/v could not be matched by that of any concentration of acetonitrile or methanol (Supporting Information Figure S1). It is noteworthy that 1,2-hexanediol was also found to be applicable for compounds with low hydrophobicity (Log *P* <1.0) when used at concentrations as low as 0.5–1.0% v/v, matching 10–20% v/v methanol (Supporting Information Figure S2).

The ability of 1,2-hexanediol to elute highly hydrophobic compounds at low concentrations can be explained by its high hydrophobicity as it has a Log *P* value of 0.7, which is remarkably higher than that of commonly employed solvents (Supporting Information Table S1). Furthermore, 1,2-hexanediol has an amphiphilic structure with a long hydrocarbon chain and two adjacent polar hydroxyl groups. This has two consequences. First, 1,2-hexanediol can efficiently compete with the hydrophobic analytes and strongly adsorb onto the hydrophobic C18 stationary phase, which in turn results in a decrease in the overall hydrophobicity of the latter due to coating with the adjacent hydroxyl groups in 1,2-hexanediol. This adsorption of 1,2-hexanediol and the resulting availability of hydrogen bonding with the coated reversed phase would be expected to influence chromatographic selectivity. Indeed, the peak order with 1,2-hexanediol was different from that with methanol and acetonitrile and similar to that with the more structurally related isopropanol (compare peaks c and b in Figure 2). This influence of 1,2-hexanediol on chromatographic selectivity was also observed in a previous report where 1,2-hexanediol was used as a modifier to alter selectivity in electrokinetic chromatography.^34^ Second, the amphiphilic structure of 1,2-hexanediol enables micelle formation, which can enhance solubilization of the hydrophobic analytes in the mobile phase and the elution strength. Indeed, we observed a change in the slope for the curve depicting the relationship between Log *C* and Log *k* around a concentration of 8–9% v/v of 1,2-hexanediol (Supporting Information Figure S3), which was found to be commensurate with the previously reported critical micelle concentration (cmc) of 1,2-hexanediol (0.7 M).^35^

It is notable that 1,2-hexanediol has a markedly higher viscosity (87 mPa s at 20 °C) relative to that of the other commonly employed eluents (Supporting Information Table S1). However, the viscosity of its aqueous mixtures drops sharply with temperature.^36^ We therefore recommend operating at a column temperature of ≥45 °C to ensure compatibility with standard 4.00 × 10^7^ Pa (400 bar) pumps at a chromatographic column length of 250 mm. At 30% v/v 1,2-hexanediol and a mobile phase flow rate of 1.0 mL min^–1^, the resulting backpressure using a reversed-phase (C18) column with a length of 250 mm, 4.6 mm i.d., and 5 μm particle size (Phenomenex Synergi Fusion-RP) was 2.80 × 10^7^ Pa (280 bar) at 50 °C column temperature. Supporting Information Figure S4 illustrates the backpressure profile of aqueous mixtures of 1,2-hexanediol in comparison with those of methanol.

### 3.3 Tolerability and Plasma Stability

Under standard conditions (i.e., without oxygen as the optional gas, platinum cones, or flow splitting/postcolumn dilution) and the standard instrumental setup (including a standard 2.5 mm i.d. plasma torch and an AriMist nebulizer) and using a 2.1 mm i.d. chromatographic column operated at its conventional flow rate of 0.25 mL min^–1^, we observed no plasma instability throughout the study with mobile phases containing concentrations of up to 30% v/v of 1,2-hexanediol (higher concentrations were not tested), and reflected power remained constant at 0 W with an applied RF matching value of 1.3–1.8 V (depending on the concentration of 1,2-hexanediol). Prolonged operation under the above conditions did not produce a significant change in analyzer pressure or visible carbon buildup on the interface (Supporting Information Figure S5). Additionally, a plasma torch with a 1.5 mm i.d. was also tested with similar results except that no adjustment for the RF matching value was required (default value of 1.3 V was used).

Even though narrow-bore chromatographic columns are generally preferable due to reduced solvent consumption and increased sensitivity, we tested higher mobile phase flow rates frequently employed with 4.6 mm i.d. columns. At 30% v/v of 1,2-hexanediol (which provides superior elution strength to that of >90% v/v methanol or acetonitrile, as described above) and mobile phase flow rates of up to 1.5 mL min^–1^, the plasma was stable (<3 W reflected power), and the sensitivity was the highest at a total carrier gas flow rate (nebulizer gas + argon make-up gas) of 0.90 L min^–1^ including a 0.65 L min^–1^ nebulizer gas flow rate (AriMist nebulizer) and a 0.25 L min^–1^ make-up (argon) gas mobile phase flow rate (Supporting Information Figure S6). Lower organic contents were found to require a higher total carrier gas flow rate for maximum sensitivity (0.90–1.0 L min^–1^ for 5.0–30% v/v 1,2-hexanediol).

For high mobile phase flow rates (>0.75 mL min^–1^) combined with a high 1,2-hexanediol content (>25% v/v), the RF matching had to be increased gradually up to 2.2 V to maintain the reflected power at <3 W and plasma stability. It is also noteworthy that for these combinations of high mobile phase flow rates and organic content, we observed plasma instability when using certain combinations of a low nebulizer gas flow rate (<0.60 L min^–1^) and a low total carrier gas flow rate (<0.9 L min^–1^). These conditions were however practically irrelevant as they were associated with a decrease or no significant change (within ±20%) in sensitivity (e.g., see Supporting Information Figure S6). Similar patterns were observed using a micromist nebulizer, and maximum sensitivity and plasma stability were achieved at 0.90–0.95 L min^–1^ nebulizer/carrier gas flow rates (no argon make-up gas required) at 30% v/v 1,2-hexanediol and a mobile phase flow rate of up to 1.5 mL min^–1^ (higher concentrations of 1,2-hexanediol and mobile phase flow rates were not tested).

The exceptionally high tolerability of 1,2-hexanediol becomes most evident when comparing it with the other commonly used solvents in reversed-phase chromatography. At conditions comparable to the above described (including a 2.5 mm i.d. plasma torch), it was not possible to sustain a stable plasma even at mobile phase flow rates of <0.25 mL min^–1^ at concentrations >10–25% of acetonitrile, methanol, or isopropanol, which is in sharp contrast with the observed stability of 1,2-hexanediol at 30% v/v concentration and up to 1.5 mL min^–1^ mobile phase flow rate. The use of the 1.5 mm torch, which is known to confer a much higher tolerability for organic solvents, was deliberately avoided in order to demonstrate the tolerability of 1,2-hexanediol in comparison with that of the commonly used organic solvents.

It is generally known that solvents with high boiling points and low vapor pressure are better tolerated by the plasma because of the reduced carbon load due to vapor transfer. Indeed, when comparing with methanol, the current data showed that at v/v % concentrations corresponding to equal carbon molarity, 1,2-hexanediol results in roughly half the carbon load, as estimated by monitoring the ^40^Ar ^12^C signal (Supporting Information Figure S7). A thorough discussion of the criteria contributing to a higher plasma tolerance for organic solvents can be found elsewhere.^13^

### 3.4 Influence on Sensitivity

**Figure 4:**
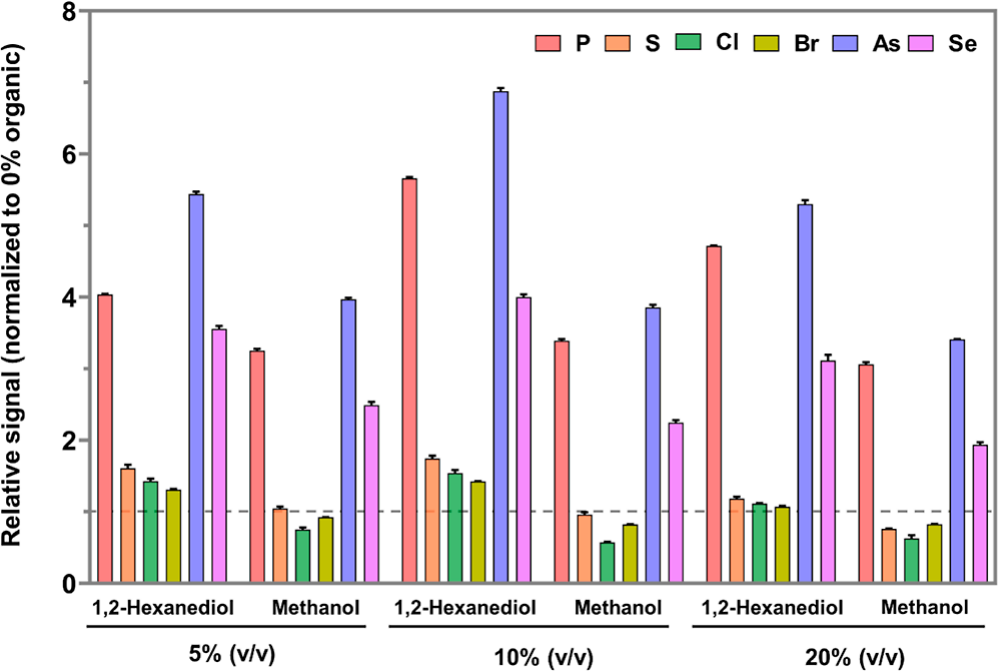
Influence of 1,2-hexanediol and similar concentrations of methanol on the sensitivity for the detection of multiple elements (in inorganic forms) relative to pure water. The *y*-axis shows the signal enhancement/suppression factors calculated based on the signal response ratio between the investigated composition of the organic solvent and 100% aqueous solution (all with 0.1% formic acid) for inorganic forms of the investigated elements injected onto the reversed-phase chromatographic column. The error bars represent the standard deviation (*n* = 3). Attention has to be paid to the difference in carbon molarity between pure methanol and pure 1,2-hexanediol (25 and 48 M for methanol and 1,2-hexanediol, respectively). It is noteworthy that sensitivity in ICPMS/(MS) detection depends on not only carbon concentration but also the nebulization properties of the eluent, which is a product of several factors including viscosity, surface tension, and droplet size distribution. Therefore, while general conditions employed for these experiments were identical (see the Experimental Section), each composition of the organic solvent required applying slightly different optimum carrier gas (higher organic compositions were found to necessitate lower carrier gas flow rates).

The impact of 1,2-hexanediol and methanol on the sensitivity of detection for six commonly involved elements in speciation analysis using HPLC-ICPMS/MS was compared within the concentration range of 5.0–20% v/v (note that the carbon molarity values in pure 1,2-hexanediol and pure methanol are 48 and 25 M, respectively). It was found that 1,2-hexanediol resulted in higher sensitivity than methanol when normalized to 100% aqueous media (Figure 4). Overall, 1,2-hexanediol at concentrations of up to 20% v/v was found to enhance the signal for all elements tested (relative to 100% aqueous solution) including a slight enhancement for chlorine, which among the tested elements has the highest ionization potential at 13.0 eV (Figure 4). By contrast, in order to match the elution strength of the above concentrations of 1,2-hexanediol, up to 90% v/v methanol would be needed, which would require employing the organic ICPMS mode and therefore significantly compromise sensitivity. The limits of detection achievable when using a mobile phase containing 10% v/v 1,2-hexanediol were estimated based on the S/N = 3 method to be 0.1 μg P L^–1^, 0.3 μg S L^–1^, 0.01 μg Se L^–1^, 0.003 μg As L^–1^, 4.2 μg Cl L^–1^, and 2.0 μg Br L^–1^ (injection volume: 50 μL, based on the chromatographic peak for the inorganic form with a peak width of 0.3 min).

A high concentration of carbon generally suppresses the ICPMS signal, particularly for elements with high ionization energy such as the halogens.^37^ However, a few elements, notably arsenic, selenium, and phosphorous, are known to show an increase in the signal at moderate carbon concentrations through the so-called “carbon enhancement effect”,^38^ which has been previously investigated.^39^ The outcome of organic media on detection via ICPMS is however not straightforward since the final signal response is governed not only by carbon concentration but also by the impact of the presence of an organic solvent on physical properties such as viscosity and surface tension, which affect the nebulization process.

The basis of the superior sensitivity with 1,2-hexanediol compared with that of methanol is not clear. It is worth noting however that due to its amphiphilic structure, 1,2-hexanediol can act as a surfactant and greatly lower the surface tension of water. Indeed, the surface tension of 5.0–20% v/v of 1,2 hexanediol was reported to be within the range of 25–26 mN m^–1^ at 20 °C,^40^ which is considerably lower than that for corresponding concentrations of methanol (50–65 mN m^–1^).^41^ It can be assumed that lower surface tension might result in a more efficient nebulization process by producing a finer spray, which can result in not only more efficient droplet transfer to the plasma but also more rapid desolvation of the fine droplets within the plasma. The effects of surfactants on the nebulization efficiency in atomic spectrometric techniques have been previously discussed.^42^ Additionally, it is plausible that more rapid desolvation of finer droplets may have contributed to the observed high plasma stability with 1,2-hexanediol.

### 3.5 Proof-Of-Concept Applications

#### 3.5.1 Sulfur Speciation in *Allium sativum* (Garlic)

**Figure 5:**
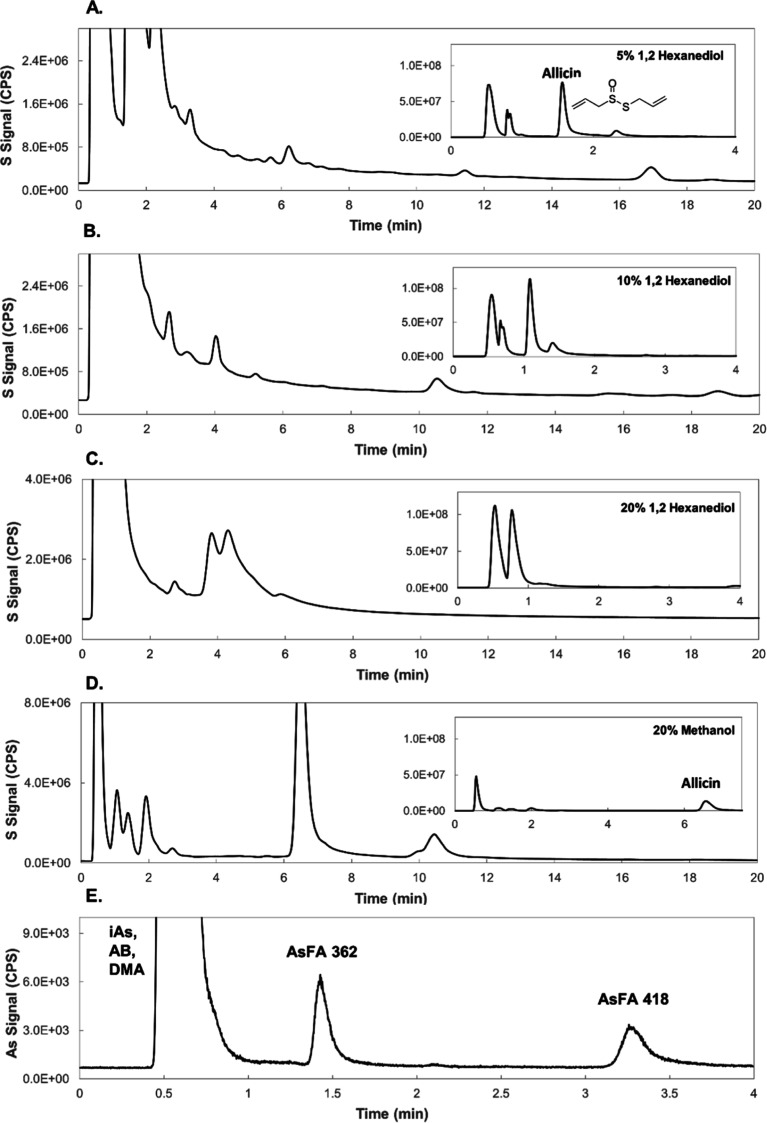
Applications involving the use of 1,2-hexanediol as an eluent for speciation analysis using HPLC-ICPMS. Chromatograms A–D show the sulfur metabolomic profile in a methanolic extract of freshly minced garlic (*Allium Sativum*) at ca. 0.5 g mL^–1^. Different concentrations of 1,2-hexanediol and methanol (indicated on the chromatograms) as eluents were used, and the resulting profiles were compared. A larger number of features was observed under 1,2-hexanediol, including at least five major compounds (20–200 mg S L^–1^), such as allicin, which is known as a dominant sulfur compound in garlic, along with >10 minor and trace compounds (0.1–1 mg S L^–1^). Chromatogram E shows the detection of two arsenic fatty acids in spiked urine at a concentration of 0.05 μg As L^–1^ (injection volume: 50 μL). Inorganic arsenic (iAs), dimethylarsinate (DMA), and arsenobetaine (AB) elute in the front.

A methanolic extract of freshly minced garlic (ca. 0.5 g mL^–1^) was analyzed following direct injection into the HPLC-ICPMS/MS system, and the sulfur metabolomic profiles were compared between those resulting with 5.0–20% v/v 1,2-hexanediol and that with 20% v/v methanol (higher methanol concentrations extinguished the plasma under the standard conditions and instrumental setup employed). The major sulfur species detected was allicin at 190 mg S L^–1^ (Figure 5), which eluted at *k* = 12 with 20% v/v methanol and *k* = 3.0 with as little as 5% v/v 1,2-hexanediol. Identification was confirmed using molecular MS at *m*/*z* 163 → 41.^43^ Moreover, 1,2-hexanediol enabled the detection of a larger number of hydrophobic sulfur compounds, including at least five major compounds (20–200 mg L^–1^) and >10 minor compounds (0.1–1.0 mg S L^–1^), see Figure 5a–c. Note that the lower sensitivity under an organic ICPMS mode, which would otherwise be necessary to elute these minor compounds, may render these undetectable (Figure 5a,c). Furthermore, the simple profile under the high elution strength of 20% v/v 1,2-hexanediol over a prolonged elution time (Figure 5c) suggests that the elution of the sulfur metabolome is likely complete and renders missing yet-to-be identified compounds less likely.

#### 3.5.2 Detection of Arsenic-Containing Fatty Acids in Spiked Human Urine

The elution of arsenolipids in HPLC-ICPMS has been performed using mobile phases containing 70–100% organic solvent with detection limits typically reported in the range of 1.0–10 μg As L^–1^,^18−20^ which are considerably higher than those typically reported for low-molecular-weight hydrophilic arsenic species analyzed under standard conditions (0.005–0.030 μg As L^–1^).^21−23^Figure 5e shows the detection of arsenic-containing fatty acids with C14 and C18 carbon chains (AsFA 362 and AsFA 418) at a concentration of 0.05 μg As L^–1^ in spiked urine using as little as 10% v/v 1,2-hexanediol. Morning first-pass urine was collected from a healthy volunteer and directly injected without sample preparation other than filtration using a 0.22 μm pore size Nylon syringe filter. The calculated limit of detection for the arsenic-containing fatty acids in the spiked urine based on the S/N = 3 definition was 0.003 μg As L^–1^ (injection volume 50 μL), see Supporting Information Figure S8.

### 3.6 Safety and Environmental Aspects

1,2-Hexanediol is widely used in the cosmetic industry as a preservative with antibacterial activity as well as an emulsifying and moisturizing agent at concentrations of >2%, and its safety for human use has been investigated.^44−46^ According to current data from the European Chemicals Agency (ECHA),^47^ the toxicities of 1,2-hexanediol was tested in daphnia and microorganisms with EC10 (48 h) > 110 mg L^–1^ and EC50 (3 h) > 1000 mg L^–1^, respectively. The oral LD_50_ for 1,2-hexanediol in rats was reported to be >5000 mg kg^–1^, compared with values of 1187 mg kg^–1^ for methanol and 617 mg kg^–1^ for acetonitrile. Furthermore, 1,2-hexanediol was categorized by the European chemicals agency (ECHA) as “readily biodegradable” with 83% degradation in 28 days in a biodegradability test performed according to the OECD 301B guideline. Overall, 1,2-hexanediol appears to be less toxic than acetonitrile and methanol, and in light of the far lower concentrations of 1,2-hexanediol needed for elution, it might be considered as a greener alternative as a general eluent in reversed-phase liquid chromatography with and without ICPMS detection.

## 4 Conclusions

1,2-Hexanediol is shown to be well-tolerated by the plasma, does not negatively impact the detection sensitivity of ICPMS, and provides strong chromatographic elution of highly hydrophobic compounds at low carbon concentrations. The employment of 1,2-hexanediol in mobile phases for HPLC-ICPMS at concentrations of <30% v/v can be a replacement for >90% v/v of common organic eluents, eliminating the inconvenience and the negative impact of the organic ICPMS mode on detection sensitivity. This can increase the likelihood of detecting low levels of novel hydrophobic compounds in nontargeted analysis and enables quantification in targeted analysis at trace levels.

## Supporting Information Available

Elution of cholesterol sulfate (Figure S1), chromatographic elution of weakly hydrophobic compounds (Figure S2), change in retention over concentrations of 1,2-hexanediol spanning the cmc (Figure S3), column backpressure with 1,2-hexanediol (Figure S4), appearance of the sampler and skimmer cones with 1,2-hexanediol (Figure S5), detection sensitivity with variable mobile phase flow rates and nebulizer gas flow rates (Figure S6), comparing carbon load (^40^Ar^12^C^+^) with 1,2-hexanediol and methanol (Figure S7), detection of arsenic-containing fatty acids at 0.01 μg As L^–1^ (Figure S8), and key properties of 1,2 hexanediol and common solvents used as eluents for RP-HPLC (Table S1) (PDF)

## Dedication

The first and corresponding author wishes to dedicate this work to his newborn, Jasmin.
