# Supplementary material for: Elution with 1,2-Hexanediol Enables Coupling of ICPMS with Reversed-Pase Liquid Chromatography under Standard Conditions
Source: Anal Chem. 2022 Jun 6;94(24):8802–10. doi: 10.1021/acs.analchem.2c01769 (PMC9218959; doi:10.1021/acs.analchem.2c01769)
Supplement: Supplementary file 1 — ac2c01769_si_001.pdf [file ac2c01769_si_001.pdf]

Supporting information

Elution with 1,2-hexanediol enables coupling ICPMS with reversed-phase liquid chromatography under standard conditions

Bassam Lajin*[1], Jörg Feldmann[2], Walter Goessler[1]

Correspondence to: bassam.lajin@uni-graz.at

[1] Institute of Chemistry, Analytical Chemistry for the Health and Environment, University of Graz, Universitaetsplatz 1, 8010 Graz, Austria

[2] Institute of Chemistry, TESLA (Trace Element Speciation Laboratory), University of Graz, Universitaetsplatz 1, 8010 Graz, Austria

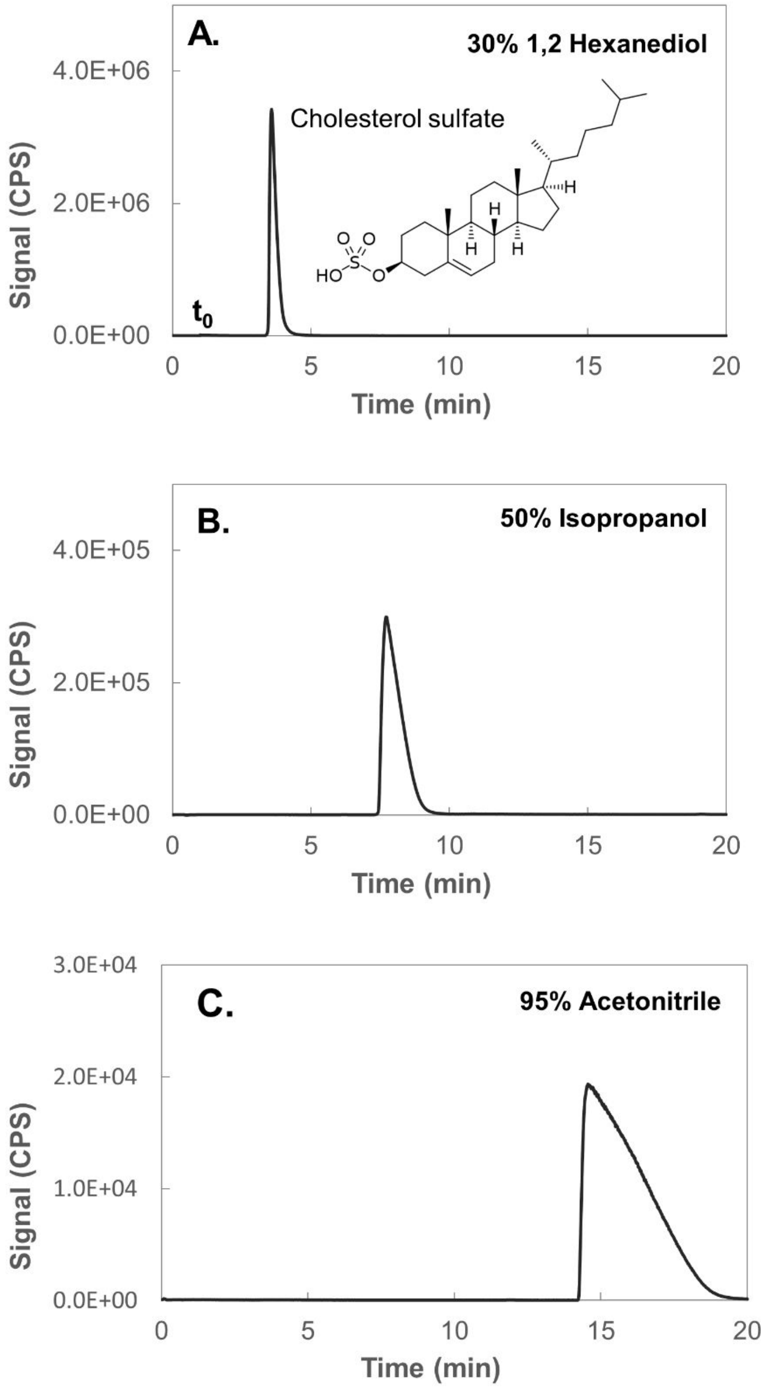

Figure S1 The elution of cholesterol sulfate under various compositions of organic solvents, namely, 1,2-hexanediol (A), isopropanol (B), and acetonitrile (C). Reasonable retention (i.e. $k<20$) was not achievable using any concentration of methanol or acetonitrile. Comparable elution strength to 30 % 1,2-hexanediol was achievable using 60 % v/v isopropanol. Due to the lack of a sufficiently strong chromophore in cholesterol sulfate and to confirm the observed elution patterns for this compound, detection was undertaken with a molecule-selective ESI-MS/MS detector in the negative mode using the mass transition 465 → 97. The column void time was 0.55 min.

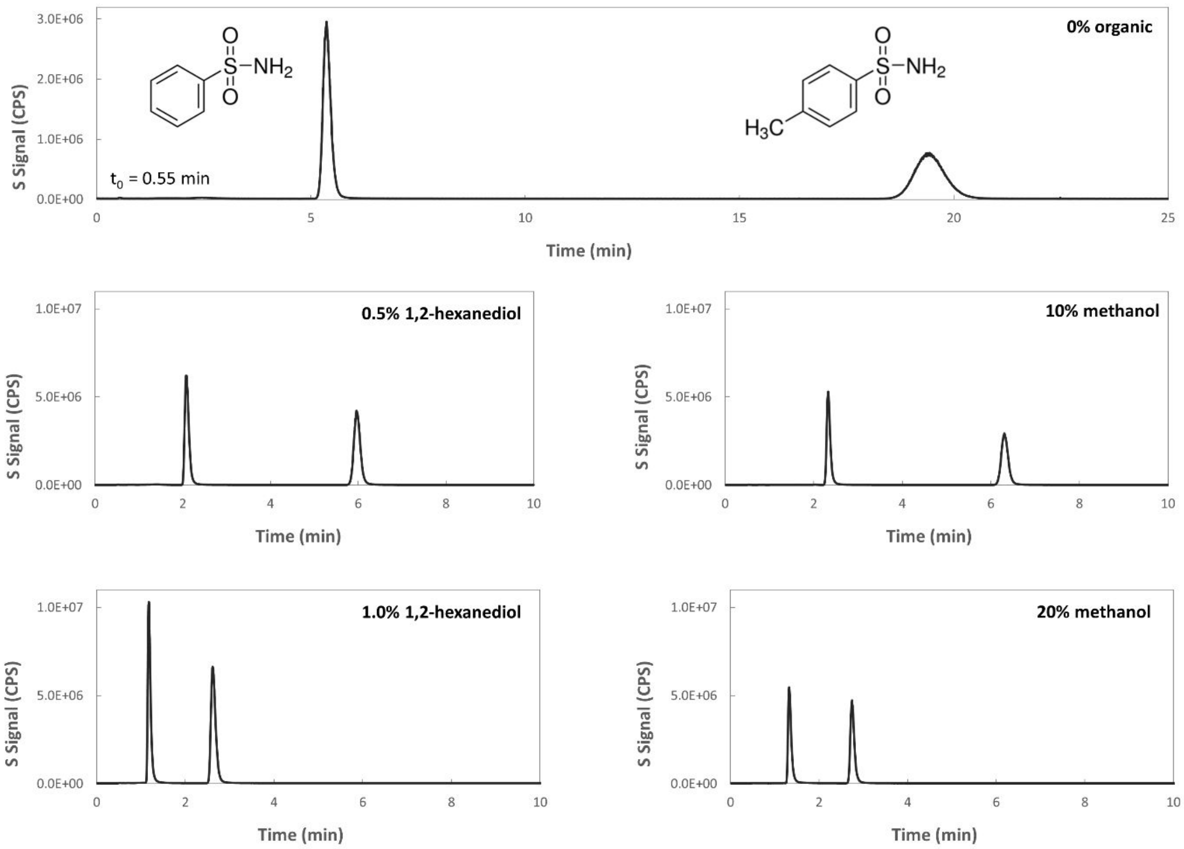

Figure S2 The elution of benzene sulfonamide (LogP 0.3) and toluene sulfonamide (LogP 0.8) with low organic proportions from the C18 reversed-phase column. The chromatograms show the elution with 100 % aqueous mobile phase as well as 0.5-1.0 % v/v 1,2-hexanediol and 10-20 % v/v methanol. Detailed chromatographic conditions can be found in the Experimental section.

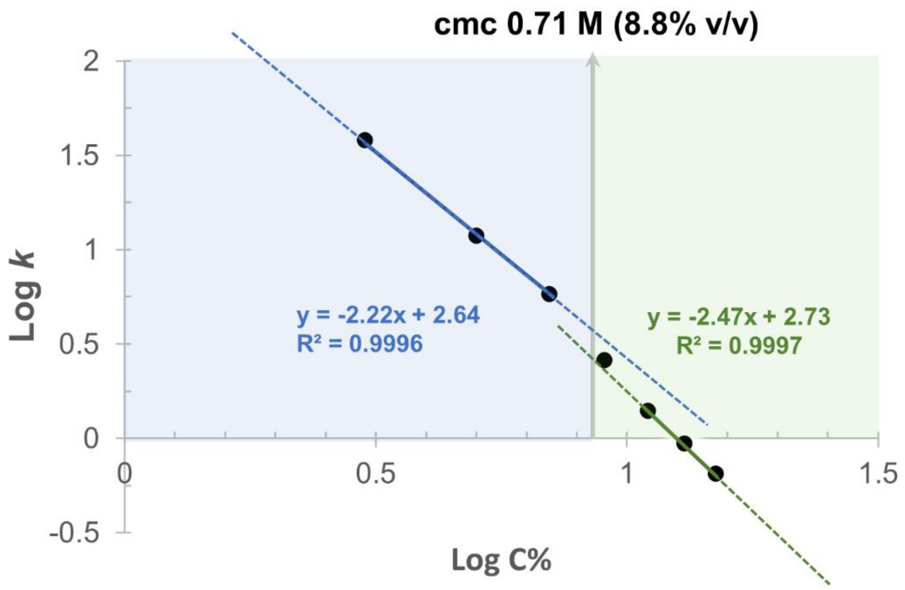

Figure S3 The relationship between retention factor and various concentrations of 1,2-hexanediol (3.0-15 % v/v) spanning the previously reported critical micelle concentration (cmc) of 0.7M (ca. 8.8 % v/v). Cloxacillin was chosen for this investigation as it maintained reasonable retention over a concentration range spanning cmc. Linear regression based on the entire dataset yielded $r^2 = 0.9955$. An increase in slope (ca. 10 %) corresponding to decreased retention by ca. 30 % is evident around the composition corresponding to cmc, which can be explained by the influence of the formation of micelles on the elution strength.

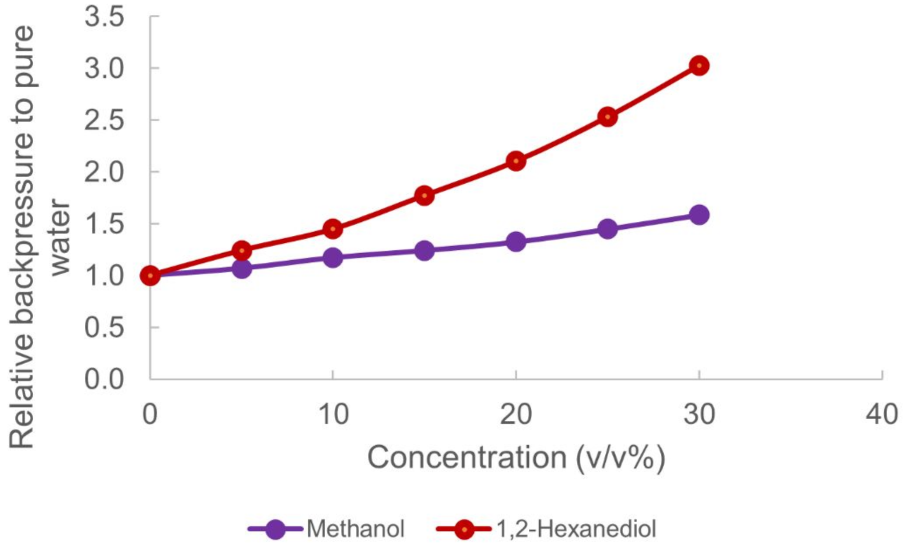

Figure S4 Comparing the backpressure under eluents containing 1,2-hexanediol with those containing methanol at comparable concentrations. The y-axis shows normalized backpressure values relative to 100 % aqueous eluent. Isopropanol showed similar patterns to 1,2-hexanediol (e.g.2.5-fold relative to pure water at 30 % v/v isopropanol). Note that this investigation was performed at 50 °C (for other chromatographic conditions see Experimental).

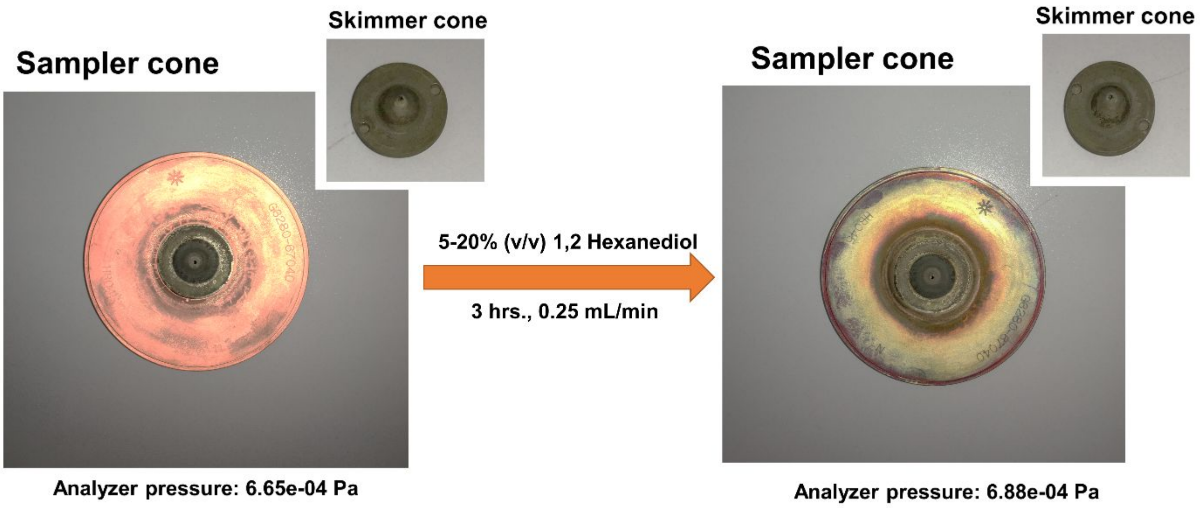

Figure S5 The appearance of the sampler and skimmer cones of the ICPMS/(MS) system following 3 hours of operation under various concentrations of 1,2-hexanediol (up to 25 % v/v) as an HPLC eluent at 0.25 mL min^{-1} flow rate. Note that the photos on the left were taken directly after cleaning the cones with a solution containing 1 % nitric acid under ultrasonication for 10 min. The discoloration that appears on the right-side photos also takes place without introducing an organic solvent. No black carbon build-up at the tips of the sampler and skimmer cones following exposure to 1,2-hexanediol was observed. The cones maintained the appearance shown for the entire study period.

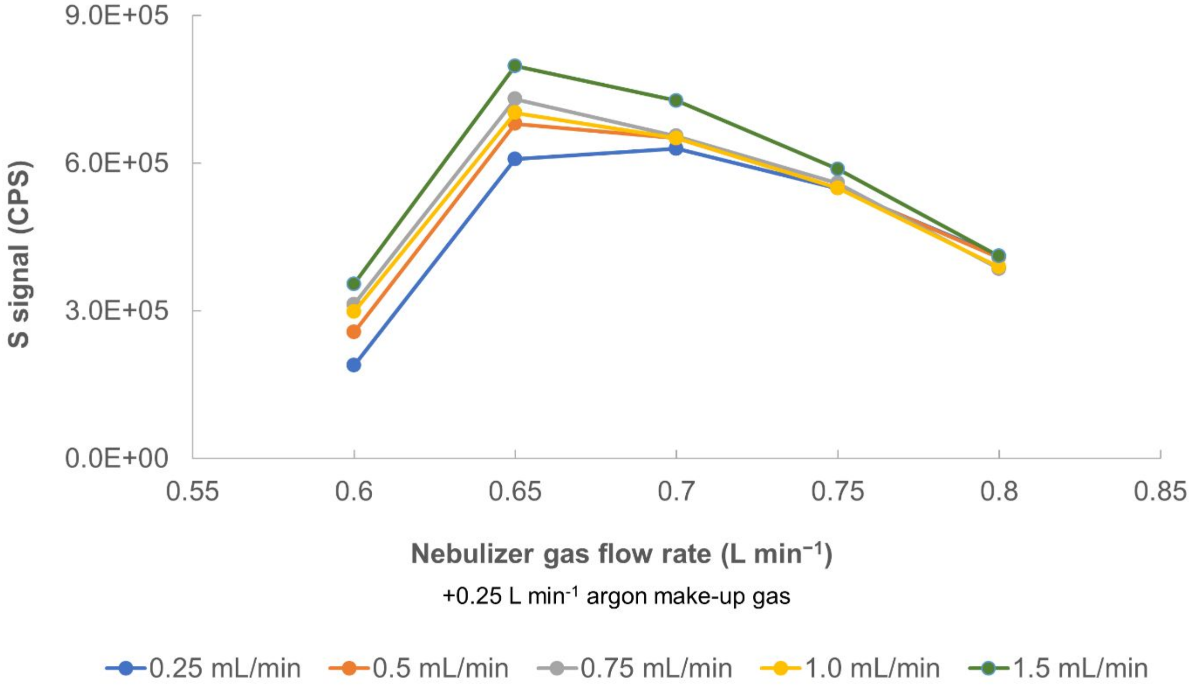

Figure S6 Investigating the plasma stability and sensitivity with 1,2 hexanediol at 30 % v/v. The employed mobile phase solution contained1,2-hexanediol at 30 % v/v in water. Five mobile phase flow rates were tested (see graph). A make-up (argon) gas fixed at 0.25 L min^{-1} was used in all experiments. Nebulizer gas flow rate was lowered gradually starting from 0.80 L min^{-1} and the RF matching increased to yield a reflected power <3 W. Note that these experiments were performed with the AriMist® nebulizer (max. operatable nebulizer gas flow rate 0.8 L min^{-1}) and the 2.5 mm torch, as described in the experimental section. Similar experiments using different values for the make-up argon gas flow rate in the range of 0.30-0.40 L min^{-1} yielded similar patterns (i.e. peak sensitivity around 6.5-8.0 × 10^5 CPS at 0.9 L min^{-1} total carrier gas flow rate), except that some combinations of high mobile phase flow rates (≥0.75 mL min^{-1}) and low nebulizer gas flow rates (<0.6 L min^{-1}) and total carrier gas flow rate (<0.9 L min^{-1}) resulted in reflected power spikes and plasma instability. These combinations were however associated with either decreased or no significant change (within ±20 %) in sensitivity.

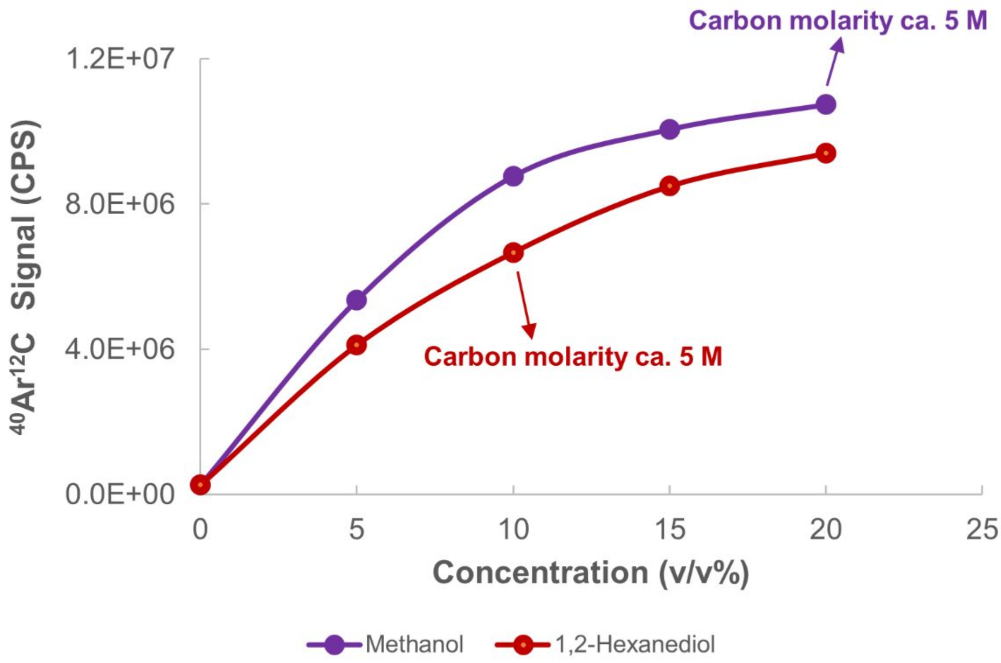

Figure S7 Comparing the plasma carbon load based on the signal of the polyatomic species $^{40}Ar^{12}C$ between methanol and comparable concentrations of 1,2-hexanediol at a mobile phase flow rate of 0.25 mL min^{-1}. The ICPMS/(MS) was operated in the no-gas mode. Note the significantly lower $^{40}Ar^{12}C$ signal for 1,2-hexanediol relative to methanol despite the fact that the former has roughly double the carbon molarity (carbon molarity in pure methanol and 1,2-hexanediol is 25 and 48 M, respectively).

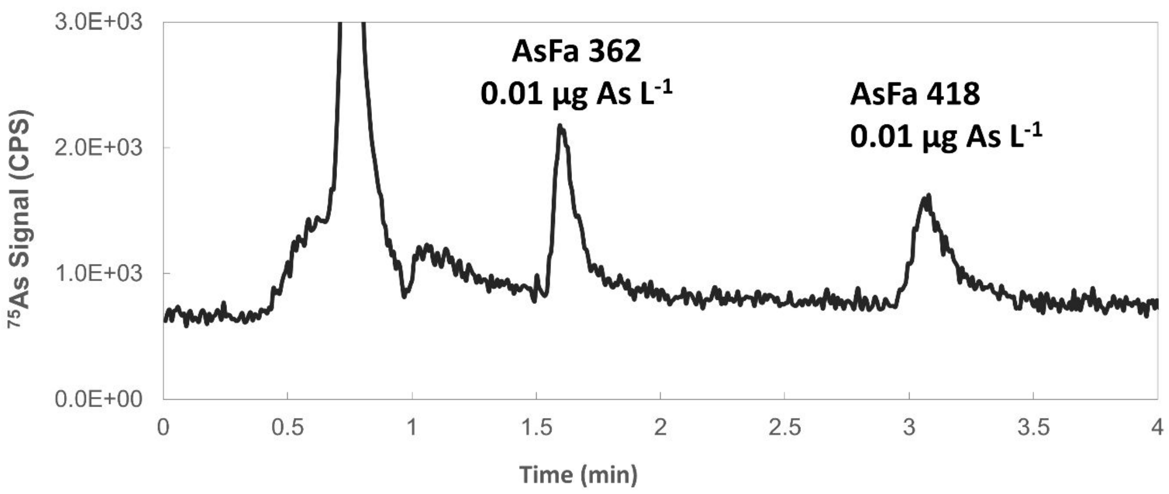

Figure S8 The detection of arsenic fatty acids 362 and 418 at 0.01 µg As L^{-1} in water. The mobile phase contained 10 % v/v 1,2-hexanediol and 0.1 % formic acid. The injection volume was 50 µL.

Table S1. Key properties of 1,2 hexanediol and common solvents used as eluents for RP-HPLC

	1,2 Hexanediol	Methanol	Isopropanol	Acetonitrile
**ICPMS – relevant parameters [a)]**				
**Boiling point (°C)**	224	65	82	82
**Density at 25 °C (g/mL)**	0.951	0.792	0.785	0.787
**Viscosity at 20 °C/50 °C (mPa s)**	87/16	0.54/0.37	2.0/0.9	0.35/0.24
**Vapor pressure at 20 °C (Pa)**	2.7	17×10^3	6.0×10^3	12×10^3
**Carbon molarity [b)]**	48	25	39	38
**HPLC – relevant parameters**				
**LogP [c)]**	0.7	-0.5	0.3	0
**H-bond donor count**	2	1	1	0
**H-bond acceptor count**	2	1	1	1
**Water miscibility (v/v%)**	Fully miscible	Fully miscible	Fully miscible	Fully miscible
**Relative column back pressure at 10/20/30% organic (50 °C) [d)]**	1.4/2.1/3.0	1.3/1.4/1.6	1.4/1.9/2.5	1.1/1.1/1.0
**Average concentrations yielding comparable elution strengths [e)]**	10 %/20 %	60 %/76 %	25 %/ 42%	40 %/63 %
**Safety**				
**Toxicity as LD_{50} (mg/kg)**	>5000	1187–2769	5045	450-787
**Chemical safety**	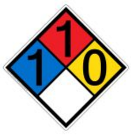	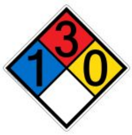	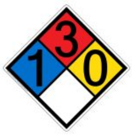	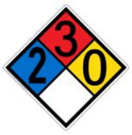

[a)] Data retrieved from PubChem and Chemspider on 28.01.2022

[b)] Calculated based on the molecular weight and density

[c)] Computed by XLogP3 3.0 [ref. 18 in the manuscript]

[d)] Column pressure ratio between 100% water and 10/20/30% organic solvent in water (determined inhouse).

[e)] average values based on five compounds (see manuscript)
